# Goblet cell carcinoma of appendix detected during surgery for ovarian tumors

**DOI:** 10.1002/ccr3.4784

**Published:** 2021-09-05

**Authors:** Rieko Kawase, Shunji Suzuki

**Affiliations:** ^1^ Department of Obstetrics and Gynecology Nippon Medical School Tokyo Japan

**Keywords:** diagnosis, goblet cell carcinoma of appendix, mucinous ovarian tumor

## Abstract

An appendectomy with a thorough examination of the gastrointestinal tract should be performed in women with a mucinous ovarian tumor.

## Graphical abstract

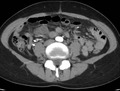

Goblet cell carcinoma is usually diagnosed after simple appendectomy; however, the current case was detected during surgery for ovarian tumors.

A 51‐year‐old female patient was referred to our institute because of an ovarian tumor from a health screening clinic. Computed tomography scan revealed a multilocular ovarian cyst of 13 cm in diameter with irregular wall thickening. The appendix was normal (0.6 × 5.5 cm, Figure 1). All blood tests were normal including carcinoembryonic antigen.

**FIGURE 1:**
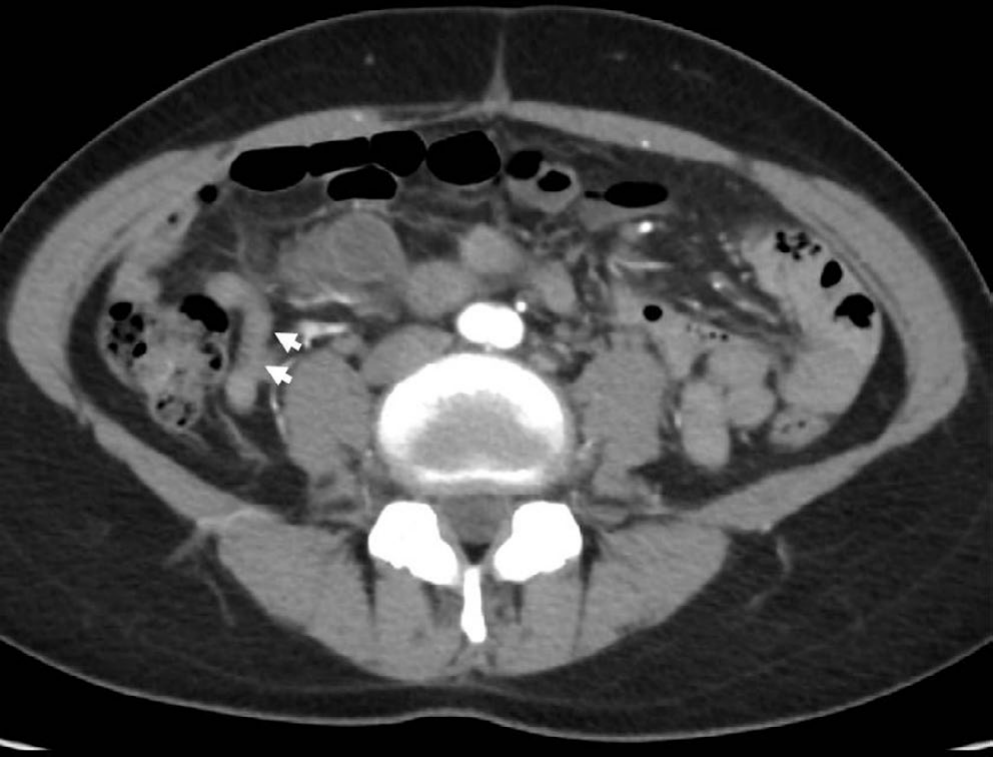
Appendix was normal based on computed tomography (0.6 × 5.5 cm, white arrows)

Left salpingo‐oophorectomy and rapid pathologic diagnosis were performed. A mucinous carcinoid tumor was revealed possibly being a primary tumor or metastasis of gastrointestinal cancer. On additional search of the aspect of peritoneum and gastrointestinal tract, an induration was palpable in the appendix. Since the rapid pathologic result of the removed appendix was goblet cell carcinoma (GCC; Figures 2 and 3), hysterectomy, right salpingo‐oophorectomy, omentectomy, and ileocecal resection were added according to the guidelines of the Japan Society of Gynecologic Oncology (https://jsgo.or.jp/). No malignant cells were detected in other organs. She has been in good condition.

**FIGURE 2:**
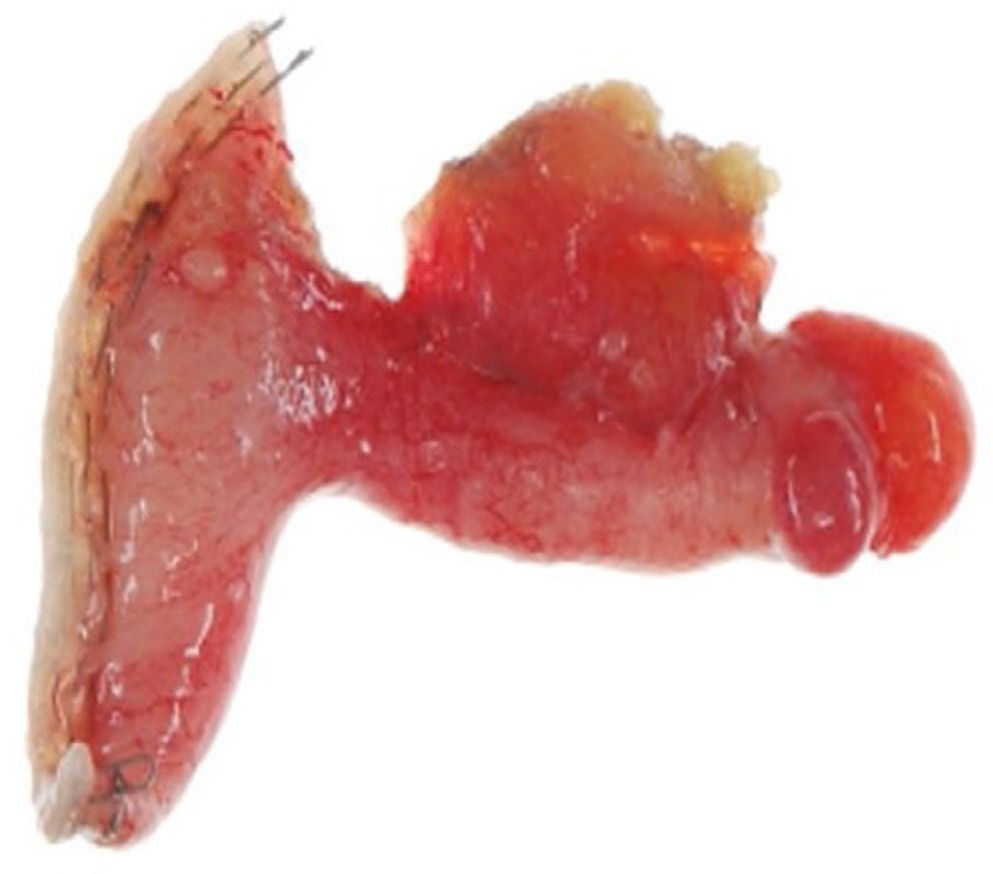
Macroscopic findings of the appendix: 0.8 × 4.5 cm, thickening of the appendix wall was observed

**FIGURE 3:**
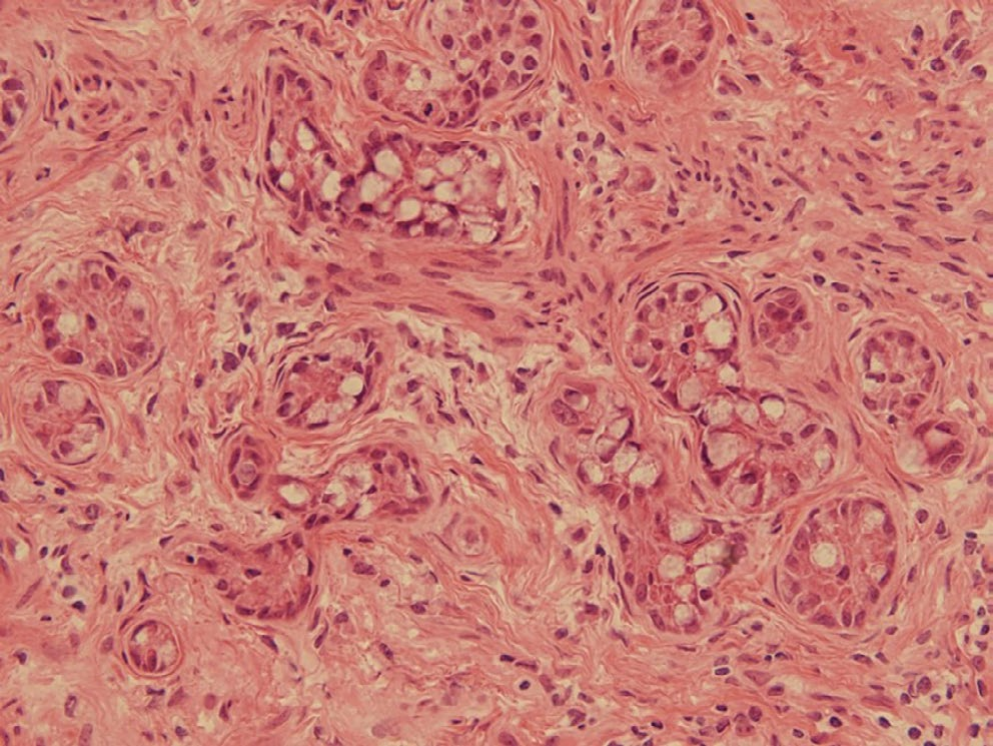
Pathologic findings of the appendix: diffuse infiltration of goblet tumor cells into the periappendiceal fat

Goblet cell carcinoma is a distinct clinical and pathologic entity with variable malignant potential. Appendiceal neoplasms with ovarian metastasis were considered to be rare; however, bilateral oophorectomy has been recommended in cases of GCC.^1^ On the other hand, an appendectomy should be performed in women with mucinous ovarian tumors or suspected GCC.^2^

## CONFLICT OF INTEREST

All authors declare no conflict of interest relevant to this article.

## AUTHOR CONTRIBUTIONS

RK (primary author) analyzed the data and wrote the manuscript. SS involved formulating the concept of the study, analyzed the data, and drafted the manuscript.

## ETHICAL APPROVAL

Prior to the case study, written informed consent was obtained from the patient. This case report was approved and licensed by the Ethics Committee of the Nippon Medical School.

## Data Availability

The data that support the findings of this study are available on request from the corresponding author. The data are not publicly available due to privacy or ethical restrictions.
